# Wax ester profiling of seed oil by nano-electrospray ionization tandem mass spectrometry

**DOI:** 10.1186/1746-4811-9-24

**Published:** 2013-07-06

**Authors:** Tim Iven, Cornelia Herrfurth, Ellen Hornung, Mareike Heilmann, Per Hofvander, Sten Stymne, Li-Hua Zhu, Ivo Feussner

**Affiliations:** 1 Albrecht-von-Haller-Institute for Plant Sciences, Department of Plant Biochemistry, Georg-August-University, Justus-von-Liebig-Weg 11, 37077 Göttingen, Germany; 2 Department of Plant Breeding and Biotechnology, Swedish University of Agricultural Sciences, Box 44230-53 Alnarp, Sweden

**Keywords:** Jojoba seed oil, Lipid profiling, Wax ester molecular species

## Abstract

**Background:**

Wax esters are highly hydrophobic neutral lipids that are major constituents of the cutin and suberin layer. Moreover they have favorable properties as a commodity for industrial applications. Through transgenic expression of wax ester biosynthetic genes in oilseed crops, it is possible to achieve high level accumulation of defined wax ester compositions within the seed oil to provide a sustainable source for such high value lipids. The fatty alcohol moiety of the wax esters is formed from plant-endogenous acyl-CoAs by the action of fatty acyl reductases (FAR). In a second step the fatty alcohol is condensed with acyl-CoA by a wax synthase (WS) to form a wax ester. In order to evaluate the specificity of wax ester biosynthesis, analytical methods are needed that provide detailed wax ester profiles from complex lipid extracts.

**Results:**

We present a direct infusion ESI-tandem MS method that allows the semi-quantitative determination of wax ester compositions from complex lipid mixtures covering 784 even chain molecular species. The definition of calibration prototype groups that combine wax esters according to their fragmentation behavior enables fast quantitative analysis by applying multiple reaction monitoring. This provides a tool to analyze wax layer composition or determine whether seeds accumulate a desired wax ester profile. Besides the profiling method, we provide general information on wax ester analysis by the systematic definition of wax ester prototypes according to their collision-induced dissociation spectra. We applied the developed method for wax ester profiling of the well characterized jojoba seed oil and compared the profile with wax ester-accumulating *Arabidopsis thaliana* expressing the wax ester biosynthetic genes *MaFAR* and *ScWS*.

**Conclusions:**

We developed a fast profiling method for wax ester analysis on the molecular species level. This method is suitable to screen large numbers of transgenic plants as well as other wax ester samples like cuticular lipid extracts to gain an overview on the molecular species composition. We confirm previous results from APCI-MS and GC-MS analysis, which showed that fragmentation patterns are highly dependent on the double bond distribution between the fatty alcohol and the fatty acid part of the wax ester.

## Background

The use of oilseed crops for sustainable production of high value chemicals for industry has received increasing attention in light of diminishing resources of fossil hydrocarbons [1]. Wax esters are a class of highly hydrophobic neutral lipids occurring in a variety of organisms. They serve numerous functions including carbon storage [2-5], protection against water loss, radiation and pathogen attack in the cuticular layer of plants [6], construction material of honeycombs [7], buoyancy regulation of marine organisms [8,9], a constituent of the sound transmission tissue of toothed whales [10-12] and as ingredients of gland secretions in mammals and birds [13,14]. Natural wax esters are esters of long-chain fatty acids and long-chain fatty alcohols synthesized by two enzymatic reactions. Firstly the reduction of either acyl-CoA or acyl-ACP substrates to fatty alcohols is catalyzed by the action of a fatty acyl reductase (FAR). Secondly the condensation of a fatty alcohol with a fatty acyl-CoA or acyl-ACP is catalyzed by a wax ester synthase (WS), giving rise to the wax ester molecule. Wax ester synthesis and the identification of the corresponding FAR and WS enzymes have been described for a variety of organisms including bacteria [15-17], protozoa [14,18], mammals [19,20], birds [21,22], insects [18,23] and plants [24-29]. The different wax ester sources exhibit distinct wax ester profiles. This is on the one hand caused by characteristic substrate preferences of the respective FARs and WSs from the different organisms and tissues and on the other hand by differences in the availability of acyl-CoA or acyl-ACP substrates. The diversity of enzymes should be a valuable tool box for biotechnological approaches that aim the accumulation of tailored wax esters in heterologous expression systems. As wax esters have favorable properties for industrial applications, especially for lubrication formulas, there is a need for sustainable sources of wax esters independent of the cost intensive refining of fossil hydrocarbons. In former times spermaceti oil originating from the spermaceti whale sensory organ presented the main source of industrial wax esters being predominantly composed of oleyl oleate [30], a wax ester with excellent chemical and physical properties for lubrication. Since whaling has been stopped, the only substantial natural source for wax esters is seed oil from the desert shrub jojoba (*Simmondsia chinensis*). Jojoba seed oil is composed of a variety of different wax esters predominated by wax esters harboring very long monoenoic aliphatic chains. Scientists are exploring means for an alternative source of wax esters with tailored species composition by transferring the wax ester biosynthesis to oil seed crops [28,31]. To evaluate the wax ester composition of seed oil obtained from transgenic plants, there is the need for analytical methods that enable quantitative wax ester profiling on the molecular species level. Wax ester profiling could also be highly useful for the elucidation of specific compositions of epicuticular waxes from different plant sources. These waxes have important biological functions as water barriers and interfaces for abiotic and biotic interactions. The knowledge about the composition and biosynthesis by wax ester profiling of different species and mutants could shed further light on the biology that brings about the high interspecies diversity of plant cuticular wax compositions [6]. Cuticular waxes are also exploited for industrial applications. Accordingly, Carnauba wax obtained from the epicuticular wax of the palm *Copernicia prunifera* has economical relevance as a commodity for polishes, food coatings and cosmetic products [28]. Wax ester profiling is also taken as a mean to evaluate the quality of edible oils, especially olive oil, that contains residual amounts of wax esters residing from the seed or fruit coat [32,33].

To date, the most widely used quantification method for wax esters relies on hydrolysis of wax esters and subsequent quantification of the derivatised fatty acid and alcohol moieties by gas chromatography coupled with flame ionisation detection (GC-FID) or electron impact mass spectrometry (GC-EI-MS). Due to hydrolytic separation of the alcohol and acyl moiety information about the molecular species composition is lost. The introduction of GC capillaries stable at high temperatures has paved the way for the analysis and quantification of intact wax esters by GC-MS. This has successfully been applied for the analysis of wax ester compositions from human meobium, skin surface lipids, preen gland waxes, wool wax, fennel seed oil, olive oil or human hair [32,34-39]. Detailed analysis of EI spectra from wax ester standards has provided a source for reference EI mass spectra supporting the difficult task of species identification based on mass spectra interpretation [40]. Still, high-throughput quantitative profiling of molecular species by GC-MS is hampered by the fact that unequivocal identification of wax esters with polyunsaturated aliphatic side chains from complex wax ester samples is difficult, since these species exhibit very low abundant molecular ions and low abundant diagnostic fragment ions [34,40]. A routine wax ester profiling by GC-MS is therefore difficult to achieve.

An alternative approach relies on the separation of wax esters by non-aqueous reversed-phase (NARP) high pressure liquid chromatography (HPLC) and the subsequent detection applying atmospheric pressure chemical ionization (APCI)-MS. This has successfully been applied for profiling the wax ester species composition of jojoba seed oil, bees wax, human hair wax and human tear fluid [41-44]. Profiling of skin surface lipids was also carried out combining data from RP-HPLC time of flight and triple quadrupole MS applying electrospray ionization (ESI) [45]. These studies could provide wax ester intensity profiles on molecular species level but no quantification that accounts for differences in ionization and fragmentation of the different molecular species was performed. In addition, isobaric species could only be identified by combining full scan chromatographic retention data with elaborate interpretation of fragment ion spectra from collision-induced dissociation (CID), still facing limitations on identification of polyunsaturated wax ester species [41,46]. Although NARP-HPLC separation provides retention information for wax ester identification, and peak integration results in robust quantification, the application of HPLC separation is problematic. It calls for calibration of many internal standard analyte pairs for quantitative output to account for differences in matrix effects throughout the chromatographic separation. Furthermore, since the mass data acquisition time for single species is restricted to small peak width, it asks for fast scanning MS to enable recording of many CID mass spectra in parallel. Absolute quantification by the use of an external calibration has been shown for the three main wax ester species of jojoba [43] by HPLC-MS applying multiple reaction monitoring (MRM), but a quantitative profiling method that addresses a high number of wax ester species by HPLC-MS has to our knowledge not been shown yet.

Semi-quantitative lipid analysis often relies on a so called shotgun approach that abandons chromatographic separation by direct infusion of the sample into the mass spectrometer. This facilitates the combined calibration of lipid families to one internal standard, as all analytes encounter the same matrix effect. Additionally, long data acquisition times are possible that are only limited by the stability of the ion source. Shotgun lipidomics provides a mean for semi-quantitative lipid profiling applying precursor ion or neutral loss scans and has been demonstrated as a valuable mean for the elucidation of lipid composition of a growing number of sample origins including plant, human, viral and yeast tissue [47,48]. These broad lipidomic approaches generalize ionization and fragmentation parameters of lipid groups for the generation of semi-quantitative data. A more accurate quantitative profiling can be achieved by targeted methods relying on specific mass transitions of a defined number of analytes and was i.e. successfully applied for the quantitative profiling of sterol lipids from plant tissue [49]. Direct infusion MS has also been used for the elucidation of wax ester composition of human hair wax esters [37]. They used nano-ESI with a triple quadrupole mass spectrometer applying MRM and neutral loss scanning that enabled identification of >160 human hair wax ester species. Still, the study lacks absolute quantification and, since human hair wax esters are mainly composed of saturated and monoenoic wax esters with even and odd carbon numbers, this study neglects wax esters with multiple double bonds that typically occur in plants.

Here, we present a direct infusion nano-ESI tandem mass spectrometry (nano-ESI-MS/MS) method for fast semi quantitative screening of wax ester profiles that relies on targeted MRM detection of intact wax ester species. With the established method, the wax ester profile of seeds from transgenic *Arabidopsis thaliana* that express a *MaFAR* from *Marinobacter aquaeolei*[50] and a *ScWS* from *Simmondsia chinensis* (jojoba) [25] was recorded and compared to the jojoba seed oil profile. The simple workflow for sample preparation and the short analytical time of 12 min/sample make the method applicable for fast screening approaches. This is also supported by the straight forward extraction of MRM intensity profiles, which does not require elaborate mass spectra interpretation. To enhance the sensitivity, an individual optimization of the ionization and fragmentation parameters for the specific mass transitions was performed. Furthermore, a systematic allocation of wax esters to calibration prototype groups according to their mass spectral characteristics allows semi-quantitative profiling with a reasonable calibration effort.

## Results and discussion

### Sample preparation procedure

**Figure 1:**
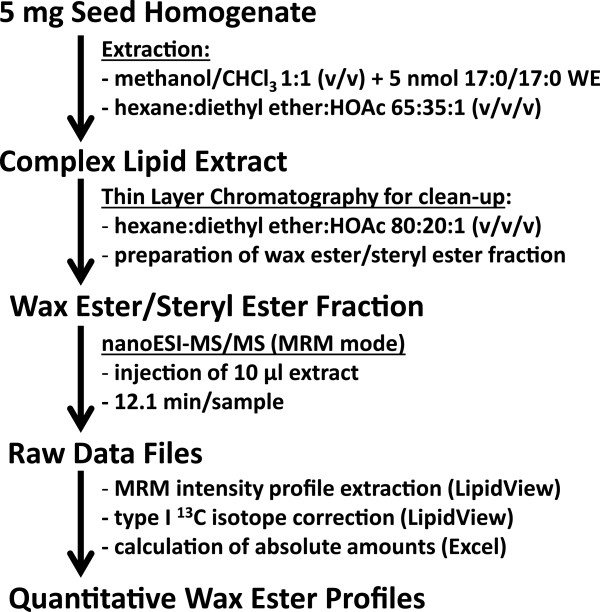
Schematic workflow of wax ester profiling by nano-ESI-MS/MS.

**Figure 2:**
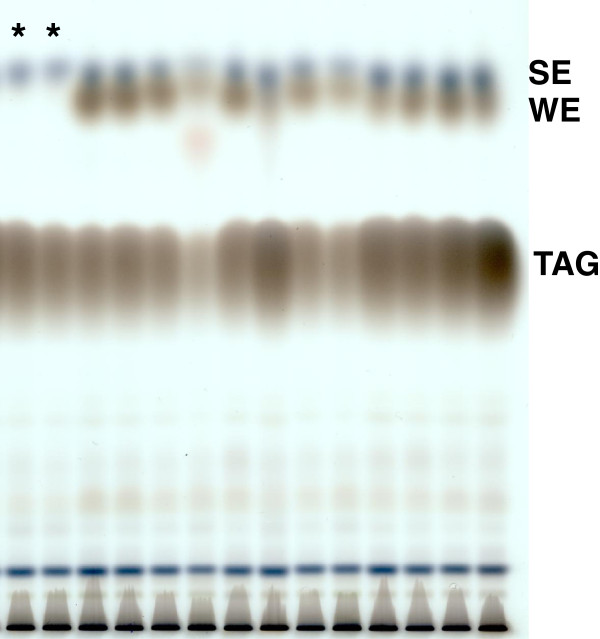
**Thin layer chromatographic separation of seed lipid extracts.** Separation of seed lipid extracts of wild type plants (*marked with asterisk) or wax ester accumulating transgenic plants. The silica TLC plate was developed with hexane:diethyl ether:acetic acid (80:20:0.1, v/v/v). Bands for the triacylglycerols (TAG), wax esters (WE) and steryl esters (SE) were identified by analysis of respective lipid class standards. Lipids were visualized by charring after immersion in cupric sulfate solution.

Seed oil was extracted from seed homogenate by organic solvents (Figure 1). To reduce the complexity of the analytical matrix, the extract was separated by TLC to obtain a purified wax ester fraction. This eliminated most of the other lipid classes from the extract, especially the predominant triacylglycerols (TAGs, Figure 2). Since the wax esters and the steryl esters cannot be separated properly by TLC, the analytical extract also contains substantial amounts of steryl esters (Figure 2). For initial experiments, the lipids were visualized by immersing the TLC plates in acidic copper sulfate and subsequent heating of the plates. Here, the lipid fractions can be discriminated by color, as the TAGs immediately turn black, while the steryl esters show a purple- and the wax esters a grey-colored band (Figure 2). To separate wax esters from other lipid classes, we applied a solvent mixture of hexane, diethyl ether and acetic acid that is widely used for wax ester preparation [31]. Variation of hexane concentrations did not enhance separation of the wax and steryl ester fraction. This observation is supported by previous publications: There are reports of separating these two ester lipid classes by applying magnesium oxide or magnesium hydroxide stationary phases [51-53], and separation by these means was for example successfully applied for wax and steryl esters of the fish parasite *Paratenuisentis ambiguus* and the moss *Dicranum elongatum*[54,55]. Unfortunately, such TLC plates are not commercially available and the need for previous separation of the steryl ester - wax ester fraction from other lipid classes under reproducible conditions makes this separation laborious. Therefore, this separation is not suitable for a routine analytical method and the combined fraction of steryl and wax esters were collected.

### Definition of wax ester prototype groups based on fragmentation behavior

The method described here aims at the quantitative profiling of plant wax esters that are defined by the possible acyl chain combinations occurring in plants. Plants commonly have an acyl-CoA pool consisting of long-chain acyl-CoAs from C16 to C24 harboring zero to three double bonds and very long-chain acyl-CoAs from C26 to C32 with saturated and monoenoic aliphatic moieties [56]. These acyl chains can either reside in the acyl or the alcohol moiety of the wax ester. Therefore, the number of possibly occurring wax esters can be up to 784 individual species. In this calculation, rare fatty acids like odd-chain and methyl-branched fatty acids are neglected, since they are not commonly found in plant waxes [57]. For quantitative profiling, a correction factor (calibration response factor, CRF) for each wax ester species has to be determined by calibration. This way, the signal intensity of a wax ester of unknown quantity can be correlated to the signal intensity of the internal standard that is supplemented in a defined amount prior to extraction. Since a calibration for every single wax ester is not feasible, we defined wax ester subgroups for combined calibration that have similar ionization and fragmentation behavior. By defining prototype groups, the effort of calibration was reduced, as calibration curves were only prepared for representative wax esters of each prototype group, thus allowing reasonable quantification of the 784 single species.

**Figure 3:**
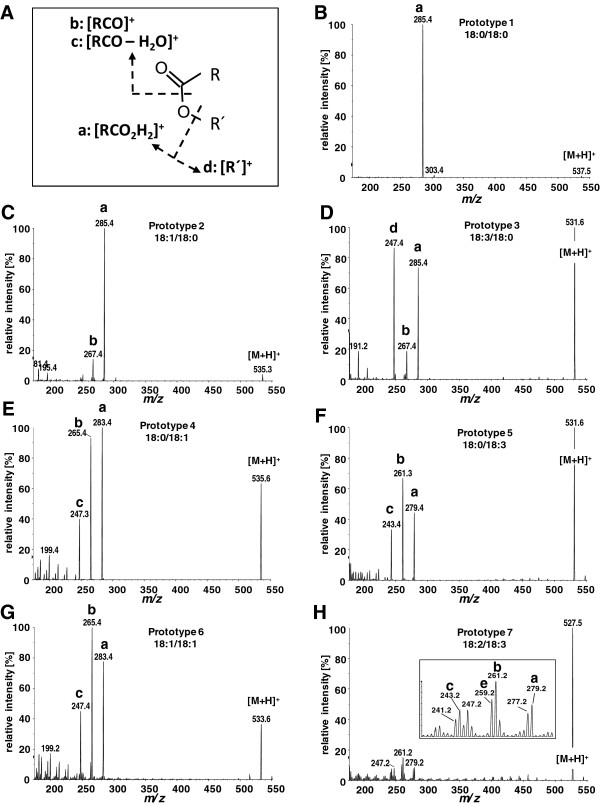
**Collision-induced dissociation mass spectra of wax ester standards.** Spectra were obtained from positive nano-ESI-MS/MS of the ammonium adduct [M+NH_4_]^+^ precursor ion representing the fragmentation pattern of seven wax ester prototype groups. Wax ester dissociate into characteristic analytical product ions **(A)** presenting the protonated acid ion a: [RCO_2_H_2_]^+^, the acylium ion b: [RCO]^+^, the dehydrated acylium ion c: [RCO – H_2_O]^+^ and the alcohol-specific loss of fatty acid fragment [R´ ]^+^ with R and R´ representing the fatty acid or the alcohol moiety, respectively. The mass spectra **(B-H)** show fragmentation patterns of wax esters as labeled. Analytical fragment ions a-d are labeled in accordance to **(A)**.

To evaluate the fragmentation behavior wax esters exhibit during direct infusion nano-ESI-MS/MS, the product ion mass spectra of 33 single wax ester standard substances were recorded, covering total carbon numbers of C36 to C48, double bond (DB) distributions at the acyl or the alcohol moiety from zero to three and total DBs of zero to six (Figure 3, Additional file 1: Table S1). Prior to the fragmentation analysis, we could show by full scan experiments in positive ionization mode that nano-ESI with an ammonium acetate-containing solvent strongly favors the formation of the ammonium adduct [M+NH_4_]^+^ over the protonated molecular ion [M+H]^+^. Therefore, the ammonium adduct was selected as the precursor ion for defining the MRM transitions. Studies of fragment ion mass spectra of wax esters obtained from GC-EI-MS analysis [34,40] showed that the fragment pattern that results from EI decomposition of the wax esters strongly depends on the distribution of DBs between the acyl- and the alcohol moiety of the molecules. This was confirmed for CID spectra obtained with APCI-MS [41]. Here, the occurrence of four major fragment ions representing the protonated acid ion [RCO_2_H_2_]^+^, the acylium ion [RCO^+^]^+^, the dehydrated acylium ion [RCO^+^-H_2_O]^+^ and the alcohol-specific fragment [R´]^+^ was described (Figure 3A). In the same line Fitzgerald and Murphy also described differences in wax ester fragmentation obtained by GC-EI-MS and nano-ESI-MS/MS experiments [37]. To probe the fragmentation behavior of wax esters during nano-ESI-MS/MS-induced decomposition, we optimized the ionization and fragmentation of 33 standard wax esters applying the automated optimization routine of the instrument software. Therefore declustering potentials, entrance potentials and collision energies for the generation of analytical fragments in the mass range from *m/z* 200 to *m/z* 500 were obtained. In this mass range the diagnostic acylium fragment ion [RCO^+^]^+^ and the protonated acid ion [RCO_2_H_2_]^+^ are found that allowed in combination with the precursor mass the unambiguous identification of the wax ester (Figure 3). In accordance with the CID spectra generated by APCI-MS [41] the formation of the dehydrated acylium ion [RCO^+^-H_2_O]^+^ and the alcohol specific fragment [R´]^+^ was also observed for the CID spectra with nano-ESI-MS/MS. From the product ion mass spectra and the fragmentation analysis, seven wax ester prototype groups were defined that show characteristic fragment ion spectra (Figure 3 B-H): For saturated wax esters the [RCO_2_H_2_]^+^ fragment occurs as the predominant diagnostic product ion (Figure 3B) allowing a sensitive detection of saturated wax esters with a high detector response for the [M+NH_4_]^+^/[RCO_2_H_2_]^+^ MRM transition (Table 1). Wax esters harboring one DB located in the alcohol moiety reveal the occurrence of a second characteristic fragment ion [RCO^+^]^+^ with [RCO_2_H_2_]^+^ being the predominant product ion for this second prototype group (Figure 3C). Wax esters with two or three DBs in the alcohol moiety and being saturated in the acyl moiety form the third prototype group. This prototype is characterized by the formation of the analytical fragment ions [RCO_2_H_2_]^+^, [RCO^+^]^+^ and the alcohol-derived fragment [R´]^+^ (Figure 3D). Product ion spectra of wax esters having one DB in the acyl moiety (prototype group four) reveal the appearance of another distinct ion representing the loss of water from the acylium ion [RCO^+^-H_2_O]^+^ (Figure 3E). Prototype group five combines wax esters harboring a saturated alcohol moiety and an acyl moiety having two or three DBs. Similar to the previous group, the fragment ions [RCO^+^]^+^, [RCO_2_H_2_]^+^ and [RCO^+^-H_2_O]^+^ are formed (Figure 3F), but here the acylium ion is formed with highest intensity. For prototype six, represented by wax esters with a monoenoic alcohol moiety and a monoenoic to trienoic acyl moiety, a similar fragmentation occurs but the intensity ratio between the protonated molecular ion [M+H]^+^ and the analytic fragment ions is clearly different to that of prototype 5 (Figure 3G). Prototype group seven combines wax esters with two or three DBs in the alcohol moiety and at least one DB in the acyl moiety. This group shows the most unfavorable fragmentation pattern. In this group, intensities of analytical fragment ions are distributed over a variety of decomposition products of which the [RCO^+^]^+^ is the most prominent one (Figure 3H). For the prototypes five to seven that can be described as polyunsaturated wax esters with at least one DB in the fatty acid part of the molecule, the acylium ion [RCO^+^]^+^ predominates the other fragment ions and is therefore taken for the definition of MRM transitions for these prototypes.

**Table 1:** Mass spectral characteristics of fourteen wax ester prototype groups

**Prototype group**				**Standards**	**Product ion**	**DP**	**EP**	**CE**	**CRF**
**#**	**C**	**DB**	**DB**						
		**OH**	**COOH**						
1	32-36	0	0	16:0/16:0	[RCO_2_H_2_]^+^	58	4.5	26	0.1753
				16:0/18:0					
				18:0/16:0					
				18:0/18:0					
2	32-36	1	0	18:1/16:0	[RCO_2_H_2_]^+^	58	4.5	26	0.0633
				18:1/18:0					
3	32-36	> 1	0	18:2/18:0	[RCO_2_H_2_]^+^	48.5	5.5	28	0.0209
				18:3/18:0					
4	32-36	0	1	16:0/18:1	[RCO_2_H_2_]^+^	52	5	23.5	0.0601
				18:0/18:1					
5	32-36	0	> 1	18:0/18:2	RCO^+^	55.5	5	23.5	0.0675
				18:0/18:3					
6	32-36	1	≥ 1	18:1/18:1	RCO^+^	43	5.5	25	0.0365
7	32-36	> 1	≥ 1	18:2/18:1	RCO^+^	43	5.5	25	0.0182
				18:2/18:2					
				18:2/18:3					
8	38-64	0	0	22:0/20:0	[RCO_2_H_2_]^+^	55	6	29	0.1482
				22:0/22:0					
				22:0/24:0					
				24:0/24:0					
9	38-64	1	0	20:1/22:0	[RCO_2_H_2_]^+^	55	6	29	0.0655
				22:1/22:0					
10	38-64	> 1	0	18:3/22:0	[RCO_2_H_2_]^+^	51	6	29.5	0.0149
11	38-64	0	1	22:0/16:1	[RCO_2_H_2_]^+^	53	5	26	0.0568
				20:0/18:1					
				22:0/18:1					
12	38-64	0	> 1	22:0/18:2	RCO^+^	50	5.5	25	0.0606
				22:0/18:3					
13	38-64	1	≥ 1	22:1/22:1	RCO^+^	52.5	6.5	27	0.0317
				20:1/20:1					
				22:1/20:2					
14	38-64	> 1	≥ 1	20:2/22:1	RCO^+^	52.5	6.5	27	0.0119
				20:2/20:2					

*Abbreviations: C* total carbon number, *DB OH* total double bonds in fatty alcohol moiety, *DB COOH* total double bonds in fatty acid moiety, *DP* Declustering potential, *EP* Entrance potential, *CE* Collision energy, *CRF* Calibration response factor.

The formation of characteristic product ion spectra according to the distribution of DBs among the alcohol and acyl moiety revealed the same fragmentation rules independent of the overall carbon number of the wax ester. Still, as the optimization of MS parameters showed a tendency towards decreased fragmentation and ionization of longer-chain wax esters (Table 1), we discriminate between wax esters with overall carbons C32 to C36 and very long-chain wax esters with C38 to C64. For both groups the above described MRM transitions are applied, but the values for the declustering potential, the entrance potential and collision energy are individually optimized, giving rise to a total of 14 prototype groups. For these 14 prototype groups representative standards were used (Table 1) to generate calibration response curves (Additional file 2: Figure S1).

### Definition of calibration response factors and linear ranges for quantification by preparing linear regression curves

To enable quantitative profiling, calibration curves of 33 wax ester standards were recorded by applying the optimized MS parameters and MRM mass transitions of the previously described prototype groups (Figure 3). Intensity profiles were recorded for a dilution series of analytes from 0.2 pmol to 50 nmol spiked with a constant amount of the internal calibrator heptadecanoyl heptadecanoate (17:0/17:0) of 5 nmol. The best fit linear regression of the ratio of signal intensities of the analyte and signal intensities of the internal calibrator as a function of the different amounts of wax ester gives the CRF value that can be expressed as:

$$\mathrm{CRF}\left[\frac{1}{\mathrm{nmol}}\right]=\frac{\mathrm{analyte}\left[\mathrm{cps}\right]}{\mathrm{internal}\;\mathrm{standard}\left[\mathrm{cps}\right]*\mathrm{amount}\;\mathrm{analyte}\left[\mathrm{nmol}\right]}$$

Applying a weighted least squares linear regression with a weighting factor of 1/×^2^ the standard curves in general reveal linear correlation of the intensity ratio of the analyte to the internal calibrator as a function of the molar amount of analyte from 5 pmol to 50 nmol (Additional file 1: Table S1). The linear range for quantification was estimated by taking the coefficient of determination (R^2^) as a measure of linearity and by applying a threshold for the coefficient of variation of the three replicate measurements not exceeding 20%. Including the dilution of 0.2 and 1 pmol in the best fit line significantly reduced the R^2^, and they often showed a coefficient of variation higher than 20%. Therefore, the amount of 5 pmol was defined as the lower limit of the linear range. The upper limit of the linear range is in most cases represented by the highest molar amount of the dilution series (Additional file 1: Table S1). One exception is the prototype group 8 representing the wax esters with saturated very long-chain aliphatic moieties. Here the upper limit of the linear range was determined, by considering R^2^ values for various subsets of the data, to be 100 pmol. This prototype represents the wax esters of highest hydrophobicity resulting in low solubility of these wax esters in the measurement solution. To address this problem we tried to use a higher chloroform content in the solvent mixture of methanol:chloroform (1:1, v/v) containing 5 mM ammonium acetate. This resulted in bad electrospray stability at the nano-ESI source. Therefore, wax esters of this prototype group display a reduced linear range from 5 pmol to 100 pmol. The overall linearity of the defined linear range of the different prototype groups is reflected by a high coefficient of determination (R^2^ > 0.9). As the slope of the linear regressions of prototypes of the same prototype group showed high consistency, the mean CRF was applied for quantification (Additional file 1: Table S1). The fact that prototype standards of one group show similar response factors that differ considerably from the other prototype groups confirms the selection of the subgroups for collective quantification. The Y-intersections of the best fit lines were neglected for quantification, as they ranged around zero with very low values.

Finally, one may argue against the application of calibrations obtained from experiments performed without sample matrix, since the influence of matrix effects is not accounted for, but in our experimental set-up the wax esters are purified by TLC prior to the nano-ESI-MS/MS measurement, strongly reducing matrix complexity to a situation that may be comparable to the calibration conditions.

### Specificity of detection applying a single MRM transition

To evaluate the analytical background of the described method, the wax ester profile of *A. thaliana* wild type seeds was recorded that should only show traces of wax esters originating from the seed coat. By analyzing the wax ester profile of wild type seeds, it became obvious that some MRM transitions are not specific, since they revealed some background signals due to unspecific detection of matrix compounds (Additional file 3: Figure S2). The TLC clean-up strongly reduces matrix complexity, but fails to separate the wax esters from the steryl esters. Therefore, one can conclude that most of the interfering background can be attributed to cross detection of steryl esters that typically occur in seed oil. A recent study of sterol lipid composition of Arabidopsis leaves has provided steryl ester profiles and corresponding lists of ammonium adduct precursor ion - product ion pairs [49]. By comparing these MRM transitions with the MRM transitions defined for wax ester detection, some overlap was identified (Additional file 4: Table S2). The highest background signals can be correlated with MRM transitions corresponding to the most common steryl esters detected in Arabidopsis leaves, namely 18:3-sitosterol, 18:2-sitosterol and 18:2-stigmasterol [49] (Additional file 3: Figure S2). Therefore, a background profile of a wild type or empty vector control sample may always be recorded, that is subsequently subtracted from the profiles of the wax ester accumulating transgenic samples. This background subtraction not only eliminates the false positive steryl ester background, but additionally subtracts all analytical noise detected in the control sample.

Besides this matrix-derived background, also false positive signals were observed that are inherent to wax esters, as they are caused by interfering detection of isobaric wax ester species. For isobaric species, specificity of the MRM transition at the level of precursor ion selection is not given due to identical [M+NH_4_]^+^ precursor masses. If the isobaric wax ester produces highly fragmented product ion spectra, there is high probability of overlapping fragment ions with an analytical product ion of an isobaric species. This is especially the case for polyunsaturated wax esters of prototype groups 7 and 14 (Figure 3H). Another source of false positive signals is caused by molecules that harbor 2 ^13^C isotopes (+2 isotopes) of unsaturated wax esters that result in false positive detection of wax esters with a double bond less. This is referred to as type II ^13^C isotope effect and Han and Gross (2001) proposed a correction factor for this [58]. This correction factor, however, is not applicable on data obtained by MRM-based measurements that include many isobaric species. To evaluate false positive signals caused by isobar detection and the type II ^13^C isotope effect, single dilutions of the 33 wax ester standards were measured. False positive signals were identified by applying the profiling method (Additional file 5: Figure S3). This revealed false positive signals for prototype groups 1 and 8 of less than % intensity of the true signal. The prototypes 2, 4, 5, 9, 11 and 12 caused false positive signals with intensities of up to 5%, the prototype groups 3 and 10 revealed false positive signals of up to 10%, and prototypes 6 and 13 showed false positive signals of up to 20%. Most critical were the prototypes 7 and 14, that exhibited false positive signals ranging from 20% to 70%. Therefore, the method may not be suitable for absolute quantification of low abundant species in the presence of a high abundant polyunsaturated isobaric wax ester. The problem of differentiation of isobaric lipid species and the elimination of isotope interference is intrinsic to shotgun methods as fragmentation pattern alone often cannot distinguish between isobars. By chromatographic separation, isobar discrimination was achieved for wax ester species that differ considerably in chain length distribution [41]. However, this chromatographic isobar separation is difficult to apply for the 784 molecular species considered here, since up to 20 isobaric species occur. The type II isotope effect may be resolved by combining algorithms for data analysis with chromatographic separations [59]. It may also be possible to resolve the type II isotope effect by ultrahigh mass resolution MS, however, such fourier transform ion cyclotron resonance MS instruments with resolutions of R_FWHM_ > 400 000 are still rare.

In most shotgun lipidomic approaches, another so called type I ^13^C isotope effect is discussed [58,60]. This describes the different contribution of ^13^C isotope signals to the total ion count of lipid species that differ in the total number of carbons. The quantification bias introduced by the type I isotope effect can be compensated by a correction factor. We employed a correction factor according to Han and Gross that corrects for the contribution of ^13^C isotopic peaks to the intensity of the monoisotopic peak [58]. The raw signal intensities of the MRM transitions with a given carbon number (CN) were corrected by multiplication with the correction factor α_CN_ given by:

$$\alpha_{\mathrm{CN}}=1+0.011n+\frac{0.011^{2}n\left(n-1\right)}{2}$$,

where n is the total number of carbons of the wax ester.

### Determination of the methodical variation and the extraction recovery

To determine the reproducibility and robustness of the profiling method, five extraction replicates and five replicate measurements of the seed extracts from one wax ester producing Arabidopsis line (explained in the next paragraph) were analyzed. For the ten most abundant wax esters detected the variation between the replicates was calculated in % relative standard deviation (% RSD). The % RSD of replicate extractions was below 12% and the % RSD for replicate measurements of the same extract was below 5% (Additional file 6: Figure S4). Additionally, the recovery rate of the 17:0/17:0 standard was determined by adding the standard before and after the wax ester extraction procedure of wild type Arabidopsis seeds. From five replicate extractions a mean recovery rate of 21% was determined.

### Comparing the wax ester profile of seed oil from transgenic arabidopsis to that from jojoba

**Figure 4:**
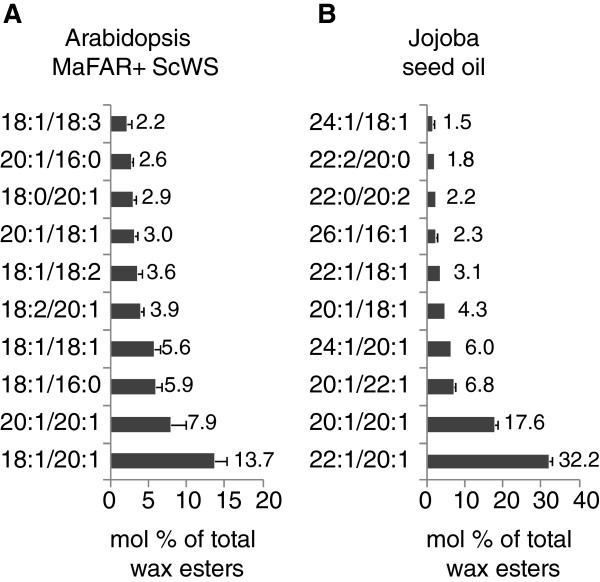
**Wax ester profiles of (A) Arabidopsis transgenic lines expressing MaFAR and JoWS and (B) jojoba seed oil.** Shown are the means (+SD) of the relative accumulation of the ten most abundant wax ester species in mol % of total wax ester accumulation of ten individual transgenic lines for **(A)** and four replicate measurements for **(B)**.

To evaluate the method, a comprehensive wax ester profile of transgenic *Arabidopsis thaliana* seeds expressing a FAR from *Marinobacter aquaeolei* (*MaFAR*) [50,61] and a WS from jojoba (*ScWS*) [25] was recorded. This profile was compared to the profile of jojoba seed oil representing the natural enzyme context of the ScWS. Transgenic T2 Arabidopsis seeds accumulated up to 70 mg wax ester/g seed. Taking into account that lipids confer around one third (36%) to the Arabidopsis seed weight [62] means that wax ester roughly make up to 20% of this seed oil fraction. In Figure 4 the background corrected profiles depicting the ten most abundant wax ester species of the seed oil from transgenic Arabidopsis and of the jojoba oil are shown, respectively. The Arabidopsis wax ester profile represents the mean of the ten profiles of individual transgenic lines, whereas the jojoba profile represents the mean of four replicate measurements. For background correction of the Arabidopsis wax ester profile the wild type profile from three replicate extractions was determined. The mean wild type wax ester profile in nmol/g seed was subtracted from the wax ester profiles of the transgenic Arabidopsis lines.

The two major wax ester species accumulating in the Arabidopsis seed oil were 18:1/20:1 accounting for 14 mol% and 20:1/20:1 accounting for 8 mol% of the overall wax ester species. In the jojoba oil profile the two species 22:1/20:1 (32 mol%) and 20:1/20:1 (18 mol%) were most abundant. The presented wax ester profile for jojoba oil is in good agreement with previously published data. It is well described that wax esters of jojoba seed oil are dominated by molecular species with even-numbered monoenoic acyl chains in each of the aliphatic moieties. Vrkoslav and colleagues measured relative wax ester profiles by comparing chromatographic peak areas of jojoba oil obtained by HPLC-APCI-MS experiments [41]. They detected wax esters with total carbons from C38 to C48 that harbored zero to three double bonds. The most abundant wax ester species were wax esters with 18:1, 20:1, 22:1 or 24:1 as alcohol moieties combined with the acyl moieties 18:1, 20:1 and 22:1 and with the wax ester 22:1/20:1 being the predominant molecular species. This data and the data presented here are also in agreement with previous quantitative profiles relying on the analysis of hydrolyzed jojoba wax esters by GC-FID and GC-MS or the analysis of selected intact species by HPLC-APCI-MS [4,43,63].

**Figure 5:**
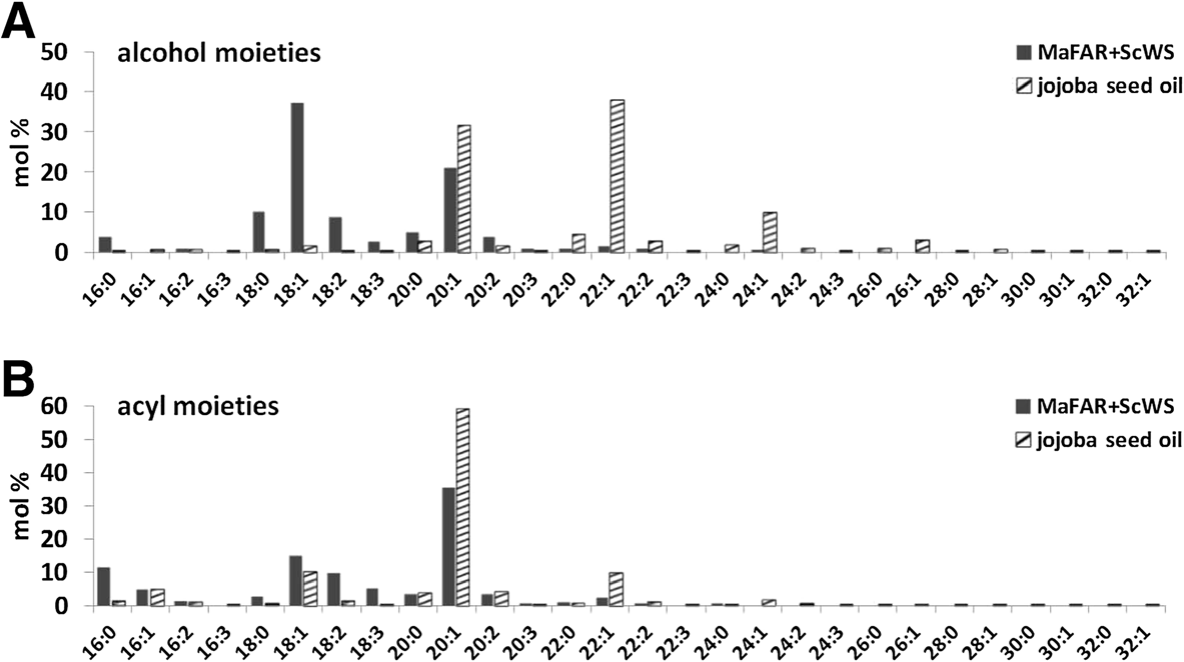
**Acyl chain profile calculated from wax ester composition.** The relative incorporation of specific acyl groups at the **(A)** alcohol moiety and the **(B)** acyl moiety that was calculated based on the wax ester profiles of transgenic MaFAR/ScWS-expressing *Arabidopsis thaliana* (MaFAR+ScWS) and jojoba seed oil.

The obtained wax ester profiles were further compared at the level of acyl moieties incorporated at the alcohol or the fatty acid part of the wax ester (Figure 5). The alcohol moiety of the jojoba oil wax esters is almost exclusively occupied by the monoenoic alcohols 20:1 (31 mol%), 22:1 (38 mol%) and 24:1 (10 mol%). The wax ester profile of the *MaFAR/ScWS*-expressing Arabidopsis reveals a high incorporation of the 20:1 alcohol (21 mol%) and of the C18-chained alcohols 18:0 (10 mol%), 18:1 (37 mol%) and 18:2 (9 mol%) that are provided for wax ester biosynthesis by the MaFAR enzyme, whereas the very long-chained alcohols with more than 20 carbons are hardly detectable (Figure 5A). Both FAR enzymes show a preference for monoenoic substrates. The different amount of alcohols formed by the two FARs is most likely not only due to differences in substrate specificity of the MaFAR preferring shorter-chained substrates compared to the ScFAR, but is also influenced by the different substrate pools in the two plant species. By comparing the incorporation of acyl-CoAs at the fatty acid position (Figure 5B) the ScWS incorporates higher levels of 20:1 and 22:1 in the natural jojoba plant background than in the transgenic Arabidopsis situation. This disagreement is most likely due to differences in acyl-CoA substrate pool availability of the ScWS in both plant backgrounds as well. The fatty acid profile of mature *Arabidopsis thaliana* Col-0 wild type seeds is composed of the following fatty acids: 16:0 (8.7%), 18:0 (3.6%), 18:1 (15.0%), 18:2 (29.0%), 18:3 (19.2%), 20:0 (2.2%), 20:1 (20.2%), 20:2 (2.0%) and 22:1 (1.7%) [62]. This profile is in good accordance with the observed acyl chain distribution of the wax esters from transgenic Arabidopsis showing the occurrence of 16:0 acyl moieties, high amounts of C18 and 20:1 acyl moieties and an almost complete lack of acyl chains longer than C20. However, most likely due to a substrate preference of the enzymes for monoenoic substrates, the 18:1 and 20:1 acyl chains show higher abundance in the wax esters of the transgenic Arabidopsis when compared to the wild type fatty acid profile. It has been shown previously for different WSs and FARs that wax ester biosynthesis is strongly influenced by the heterologous host and the availability of fatty acid substrates [28,31]. For example, this effect was demonstrated for Arabidopsis seeds that express a FAR and a WS enzyme from mouse. In Arabidopsis wild type the MmFAR1 and MmWS enzymes showed a rather broad substrate preference leading to a versatile mixture of wax ester species. However, the wax ester biosynthesis could be directed to a predominant accumulation of 18:1/18:1 wax ester by expressing the enzymes in the mutant background *fad2 fae1* that is enriched in 18:1Δ^9^-acyl CoA [15,31]. Barney and coworkers examined the specificity of five bacterial WS and could also change biosynthetic outcome by modulation of substrate availability [15].

In conclusion, by the comparison of the two wax ester profiles we could show differences in wax ester composition on the molecular species level as well as on the level of acyl chain distribution. The jojoba wax ester profile is in good accordance with the literature. From the analysis of the profiling data, it is tempting to speculate that the biosynthetic outcome of the expression of wax ester biosynthetic genes in developing plant seeds is on the one hand determined by the substrate preference of the FAR and WS enzymes and on the other hand by the availability of respective acyl-CoA substrates.

## Methods

### Standard substances

Wax ester standards were purchased from Sigma-Aldrich (Taufkirchen, Germany), Larodan Fine Chemicals AB (Gothenberg, Sweden) and Nu-Chek Prep, Inc. (Elysian, MN, USA). Long-chained unsaturated wax esters that were not available were synthesized by reacting the fatty acyl chloride with the alcohol both purchased from Nu-Chek Prep, Inc. (Elysian, MN, USA) by the following procedure: the alcohol was mixed with the acyl chloride in a molar ratio of 2 : 1 in 3 ml dried dichloromethane with an approximate concentration of 30 mg ml^-1^. The reaction was carried out by slight shaking on ice for 20 min. Addition of 10 ml water ends the reaction. The phases were separated at 450 g for 5 min and the aqueous phase was discarded. This clean-up was repeated twice. The organic phase was evaporated under streaming nitrogen and suspended in 200 μl chloroform. The wax ester was purified by TLC. The solution was spotted on a 0.25 × 20 × 20 cm F60 silica gel glass plate (Merk, Darmstadt, Germany) and developed with *n*-hexane:diethyl ether:glacial acetic acid (80:20:1, v/v/v). The wax ester band could be identified by eye and was scraped from the plate. The wax ester was recovered from the silica by extracting twice with 1 ml *n*-hexane and sedimentation of the silica at 450 g for 5 min. The typical yield of wax ester was about 1/3 of the weight of the educts. Success of synthesis and clean-up was controlled by direct infusion nano-ESI-MS Q1 full scan in a range from 50–800 Da. The jojoba oil was pure cold-pressed oil purchased from a German cosmetic supplier (Spinnrad GmbH, Bad Segeberg, Germany).

### Sample preparation

Total seed oil was extracted from 5–10 mg dry seeds. Seeds were ground with a ceramic mortar and pestle and the addition of some extra pure sea sand (Carl Roth GmbH, Karlsruhe, Germany) to a fine powder. After transferring the seed powder to a 8 ml screw lid glass tube, 2 ml chloroform:methanol (1:1, v/v) and 5 nmol of heptadecanoyl heptadecanoate internal standard were added. Lipids were extracted by shaking 20 min at 4°C. Non-soluble cell debris was pelleted by 5 min centrifugation at 450 g. The supernatant was collected in a new glass tube and the pellet was reextracted with 1 ml *n*-hexane:diethyl ether:glacial acetic acid (65:35:1, v/v/v) by shaking for 10 min at 4°C. The non-soluble cell debris was pelleted by 5 min centrifugation at 450 g and the supernatants were combined. After evaporation of the solvent under streaming nitrogen the lipid extract was dissolved in 40 μl chloroform. The crude lipid extract was spotted on 0.25 × 20 × 20 cm F60 silica gel glass plate (Merck, Darmstadt, Germany) using an automatic TLC spotter (Camag, Muttenz, Switzerland) and developed with *n*-hexane:diethyl ether:glacial acetic acid (80:20:1, v/v/v). The plate was sprayed with 8-anilino-1-naphthalenesulfonic acid (0.2%, w/v) that lipid bands could be marked under UV light. The wax ester containing band was scraped from the plate and the lipids were extracted in a glass tube twice with 1 ml *n*-hexane and sedimentation of the silica at 450 g for 5 min. The solvent supernatants were combined and evaporated under streaming nitrogen. For nano-ESI-MS/MS measurement the wax ester were dissolved in 2 ml methanol:chloroform (2:1, v/v) containing 5 mM ammonium acetate. Jojoba oil was measured without further work up in a 1:2.000 dilution.

### Generation of plant transformation vector

A plasmid (pHisMBPMaFAR) containing the gene for *Marinobacter aquaeolei* fatty acid reductase (*MaFAR*) was generated with a synthesized *MaFAR* gene and the sequence was optimized for *Escherichia coli* expression [50]. The open reading frame of *MaFAR* was then amplified by PCR using the following primer combination: *MaFAR*-*for-SalI* 5’-AGT**GTCGAC**ATGGCAATCCAGCAGGTCCAC/*MaFAR-rev-BamHI* 3’-AGT**GGATCC**TCATGCCGCTTTTTTACGTTGACG. To generate a vector with the wax ester synthesis gene (*ScWS*) from *Simmondsia chinensis* (jojoba), RNA was isolated from ripening seeds of jojoba and cDNA was obtained via RT-PCR using the following primer combination: *ScWS-for-SalI* 5’-ATG**GTCGAC**ATGGAGGTGGAGAAGGAGCTAAAG/*ScWS-rev-BamHI* 3’- ATG**GGATCC**TCACCACCCCAACAAACCCATC, based on the published sequence information [25]. The resulting PCR fragments were either cloned into pENTRY-A (*MaFAR*) or pENTRY-D (*ScWS*) and transferred into pCAMBIA33.0 via Dual-Gateway technology (Invitrogen, Carlsbad, USA) as described in Heilmann et al. [31], yielding *pCAMBIA33-pNAPIN::MaFAR_pNAPIN::ScWS*. Thus both genes are under the control of the napin promoter, which is seed specific.

### Generation of transgenic Arabidopsis lines

The transgenic Arabidopsis lines were obtained through *Agrobacterium*-mediated transformation of *Arabidopsis thaliana* ecotype Col-0 via floral dipping. For transgene selection the transformation cassette also contains a phosphinothricin selection marker. T2 seeds were harvested from T1 plants that have undergone selection for transgene expression by phosphinothricin treatment.

### Analytical setup

The analysis of wax esters was performed using an Applied Biosystems 3200 hybrid triple quadrupole/linear ion trap mass spectrometer (ABSciex, Darmstadt, Germany). Direct injection nano-ESI analysis was achieved using a chip ion source (TriVersa NanoMate; Advion BioSciences, Inthaca, NY, USA). 10 μl wax ester extract was subjected to nano-ESI in positive ionization mode with ionization voltage of 1.5 kV and backpressure of 0.4 psi. The mass analyzers were adjusted to a resolution of 0.7 amu full width at half-height. The ion source temperature was 40°C, and the curtain gas was set at 10 (given in arbitrary units). Intensity profiles of 785 wax esters (Additional file 7: Table S3) were monitored by applying multiple reaction monitoring (MRM) mode with optimized MS parameters for predefined wax ester prototype groups (Table 1). The individual mass transitions were measured with a dwell time of 150 msec in six cycles with a cycle time of 121.6805 sec/cycle. Acquisition of six cycles resulted in a total of 12.1 min/sample. As MRM precursor ion the Q1 mass analyser selected for the ammonium adduct of the wax esters [M+NH_4_]^+^ and the Q3 mass analyser selected for the [RCO_2_H_2_]^+^or the [RCO^+^]^+^ fragment ion (R represents the fatty acid moiety).

### Data collection and preparation

Peak intensities of 785 MRM transitions were collected with the Analyst 1.5.1 software (AB Sciex, Darmstadt, Germany). Signal intensities were extracted from the Analyst raw data file with the LipidView software (AB Sciex, Darmstadt, Germany) applying a target list containing the 785 MRM transitions with a mass tolerance of 0.5 amu, a minimum signal to noise value of 1 and a minimum % intensity of 0%. Type I ^13^C isotope correction of raw intensities was preformed with LipidView applying the correction factor α_CN_ as stated in the main text. Assignment of calibration response factors (CRFs) to the intensity profiles of the molecular wax ester species and calculation of absolute wax ester amounts and mol % values was performed with Excel 2007 software (Microsoft Deutschland GmbH, Unterschleissheim, Germany). To facilitate quantification, calibration curves of representative wax ester standards of each prototype group were prepared applying the MS parameters given in Table 1. Quantification was carried out using a CRF calculated from the calibration curve of intensity (*m/z*) ratios of [prototype wax ester standard]/[17:0/17:0 internal standard] *vs.* molar amounts of prototype wax ester standard (0.2 pmol-50 nmol). For the calibration, triplicate dilutions of prototype standards supplemented with 5 nmol 17:0/17:0 were analysed. For preparation of the best fit lines a weighted linear regression with a statistical weight of 1/×^2^ was applied [64] using Origin Pro 8.5 software package (OriginLab Cooperation, Northampton, MA, USA). From the best fit lines of the linear regressions we determined the CRFs. Therefore, the amount of wax ester was calculated as:

$$\begin{array}{l} \mathrm{amount}\;\mathrm{wax}\;\mathrm{ester}\left[\frac{\mathrm{nmol}}{g\;\mathrm{Seed}}\right]\\ \;=\frac{\mathrm{analyte}\left[\mathrm{cps}\right]*\alpha_{\mathrm{CN}}}{\mathrm{internal}\;\mathrm{standard}\left[\mathrm{cps}\right]*\alpha_{\mathrm{CN}}*\mathrm{seed}\;\mathrm{weight}\left[g\right]*\mathrm{CRF}\left[\frac{1}{\mathrm{nmol}}\right]} \end{array}$$

## Competing interests

The authors declare no competing interests.

## Authors’ contributions

TI and CH developed the analyical method, carried out the measurements and drafted the manuscript. PH cloned and characterized the *MaFAR* cDNA. EH generated the transgenic plants. MH cloned and characterized the *ScWS* cDNA. LHZ participated in the design of the study and performed control measurements. IF and SS conceived of the study, participated in its design and coordination and drafted the manuscript. All authors read and approved the final manuscript.

## Supplementary Material

Additional file 1: Table S1 Calibration response factors and linear ranges for 33 wax ester standards. Click here for file

Additional file 2: Figure S1 Calibration curves for prototype wax esters. The calibration curves of 33 wax ester molecular species representing 14 prototype groups are shown. Intensity profiles were recorded in triplicates for a dilution series of analytes from 0.2 pmol to 50 nmol spiked with a constant amount of the internal calibrator heptadecanoyl heptadecanoate (17:0/17:0) of 5 nmol. The linear regression correlates the intensity ratio (y-axis) of the internal standard signal in counts per second (cps) and the respective wax ester signal (cps) with a given molar amount of analyte (x-axis). For linear regression a weighted least squares fit with a weighting factor of 1/x^2^ was applied. The fit line represents the linear range according to R^2^ > 0.9 and CV< 20% (n=3). Click here for file

Additional file 3: Figure S2 Wax ester profile of *Arabidopsis thaliana* wild type seeds demonstrating false positive detection of phytosterols. The mean of the ten most abundant wax ester species in mol % of total wax esters from three extraction replicates (+SD) of wild type *Arabidopsis thaliana* seeds is shown. As a result of overlapping mass-transitions with isobaric phytosterols (Additional file 4: Table S2) the phytosterol species 18:2-sitosterol, 18:3-sitosterol and 18:2-stigmasterol lead to high false positive signals for 20:0/26:0, 20:1/26:0 and 20:0/26:1 wax ester species, respectively. Click here for file

Additional file 4: Table S2 Wax ester molecular species that are isobaric with steryl esters. Click here for file

Additional file 5: Figure S3 MRM specificity for wax ester identification. The specificity of MRM transitions for wax ester detection was probed by analyzing single dilutions of 33 wax ester standards representing prototype groups 1–14. The five false positive signals of highest intensities (cps) from wax esters not present in the sample is expressed as % ion intensity of the positive signal from the analyzed wax ester species. False positive signals originate from detection of isobaric wax ester species and the C^13^ type II isotope effect. Click here for file

Additional file 6: Figure S4 Determination of the method variation in % RSD from replicate extractions and replicate measurements. The % relative standard deviation (% RSD) from (B) five extraction replicates and (D) five replicate measurements of an individual transgenic line is shown. For the ten most abundant wax esters the mean (+SD) wax ester accumulation in mol% of total wax esters is depicted for (A) the replicate extractions and (C) the replicate measurements. Click here for file

Additional file 7: Table S3 MRM transitions with instrument parameters of all wax ester molecular species. Click here for file
