# Supplementary material for: Wax ester profiling of seed oil by nano-electrospray ionization tandem mass spectrometry
Source: Plant Methods. 2013 Jul 6;9:24. doi: 10.1186/1746-4811-9-24 (PMC3766222; doi:10.1186/1746-4811-9-24)
Supplement: Additional file 1: Table S1 — Calibration response factors and linear ranges for 33 wax ester standards. [file 1746-4811-9-24-S1.pdf]

proto-type	wax ester	linear range [pmole]	R^2	Y intercept	CRF	mean [CRF]
1	18:0/18:0	5-50.000	0.972	0.00033	0.1831	0.1753
	16:0/16:0	5-50.000	0.986	0.00042	0.1892	
	18:0/16:0	5-50.000	0.996	0.00040	0.1621	
	16:0/18:0	5-50.000	0.994	0.00006	0.1668	
2	18:1/18:0	5-50.000	0.998	-0.00003	0.0617	0.0633
	18:1/16:0	5-50.000	0.992	-0.00001	0.0649	
3	18:2/18:0	5-50.000	0.996	0.00001	0.0202	0.0209
	18:3/18:0	5-50.000	0.994	0.00001	0.0217	
4	16:0/18:1	5-50.000	0.987	0.00007	0.0579	0.0601
	18:0/18:1	5-50.000	0.997	0.00014	0.0623	
5	18:0/18:2	5-50.000	0.965	0.00009	0.0831	0.0675
	18:0/18:3	5-50.000	0.998	-0.00001	0.0519	
6	18:1/18:1	1-50.000	0.993	0.00003	0.0365	0.0365
7	18:2/18:1	10-20.000	0.991	0.00007	0.0219	0.0182
	18:2/18:2	10-20.000	0.996	0.00001	0.0160	
	18:2/18:3	10-20.000	0.997	0.00001	0.0168	
8	20:0/22:0	5-50	0.991	0.00038	0.1918	0.1482
	22:0/22:0	5-50	0.987	0.00035	0.1349	
	22:0/24:0	5-50	0.945	0.00063	0.1420	
	24:0/24:0	5-50	0.835	0.00062	0.1242	
9	20:1/22:0	5-20.000	0.962	0.00003	0.0525	0.0655
	22:1/22:0	5-20.000	0.959	0.00006	0.0784	
10	18:3/22:0	5-20.000	0.965	0.00000	0.0149	0.0149
11	20:0/18:1	5-50.000	0.977	0.00011	0.0527	0.0568
	22:0/16:1	5-50.000	0.963	0.00047	0.0603	
	22:0/18:1	5-50.000	0.926	0.00040	0.0574	
12	22:0/18:2	5-50.000	0.957	0.00009	0.0772	0.0606
	22:0/18:3	5-50.000	0.970	0.00020	0.0441	
13	20:1/20:1	5-20.000	0.971	0.00004	0.0265	0.0317
	22:1/22:1	5-20.000	0.929	0.00009	0.0247	
	22:1/20:2	5-50.000	0.942	0.00003	0.0441	
14	20:2/22:1	5-50.000	0.945	0.00003	0.0153	0.0119
	20:2/20:2	5-50.000	0.973	0.00001	0.0086	
