# Supplementary figures and images for: Wax ester profiling of seed oil by nano-electrospray ionization tandem mass spectrometry
Source: Plant Methods. 2013 Jul 6;9:24. doi: 10.1186/1746-4811-9-24 (PMC3766222; doi:10.1186/1746-4811-9-24)

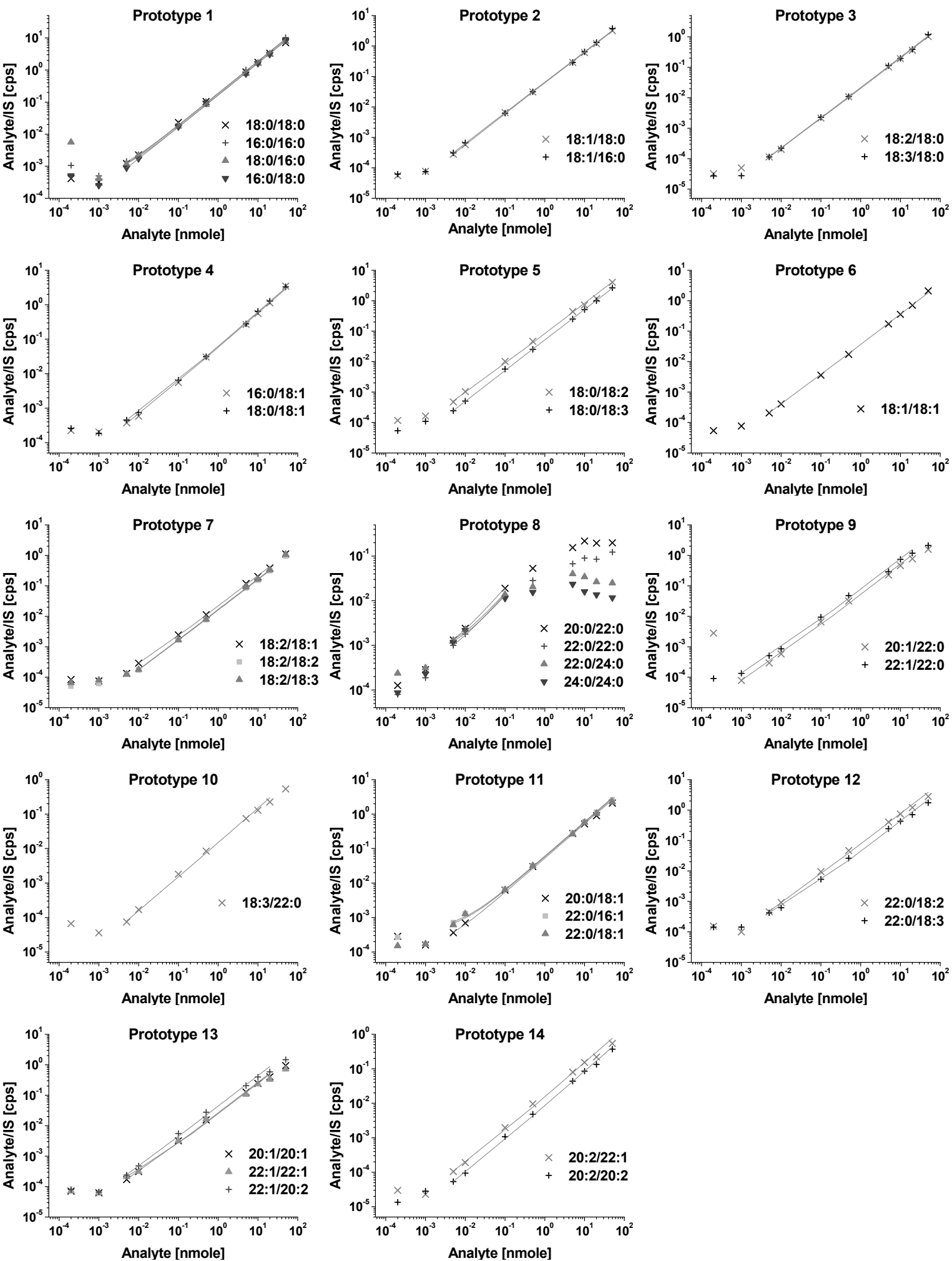

Supplement: Additional file 2: Figure S1 — Calibration curves for prototype wax esters. The calibration curves of 33 wax ester molecular species representing 14 prototype groups are shown. Intensity profiles were recorded in triplicates for a dilution series of analytes from 0.2 pmol to 50 nmol spiked with a constant amount of the internal calibrator heptadecanoyl heptadecanoate (17:0/17:0) of 5 nmol. The linear regression correlates the intensity ratio (y-axis) of the internal standard signal in counts per second (cps) and the respective wax ester signal (cps) with a given molar amount of analyte (x-axis). For linear regression a weighted least squares fit with a weighting factor of 1/x2 was applied. The fit line represents the linear range according to R2 > 0.9 and CV< 20% (n=3). [file 1746-4811-9-24-S2.pdf]

# *A. thaliana* wild type background signals

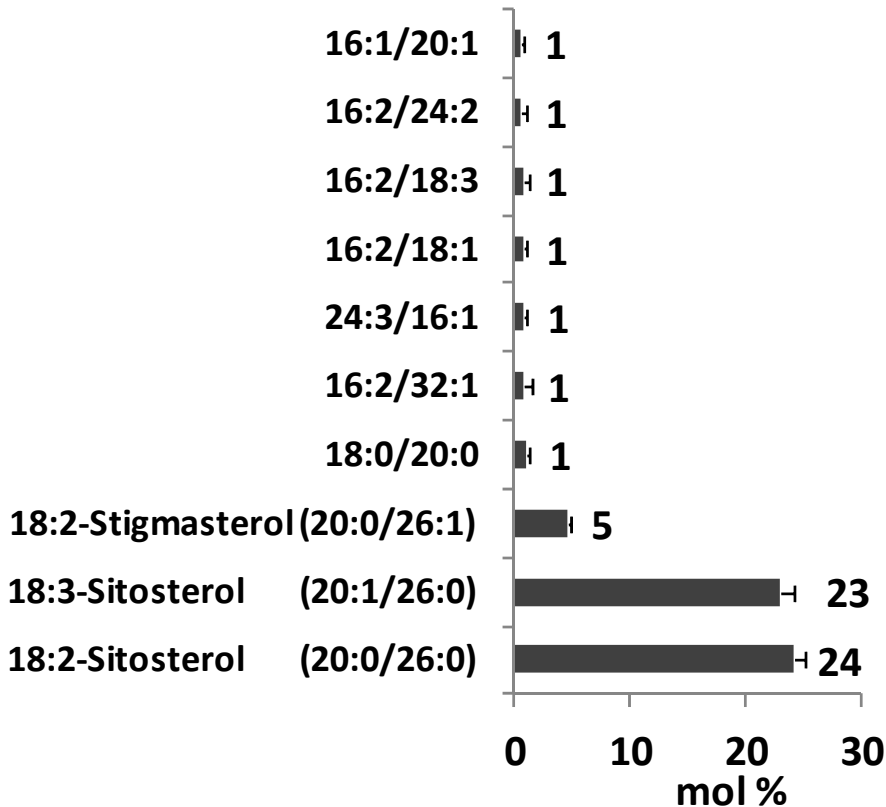

Supplement: Additional file 3: Figure S2 — Wax ester profile of Arabidopsis thaliana wild type seeds demonstrating false positive detection of phytosterols. The mean of the ten most abundant wax ester species in mol % of total wax esters from three extraction replicates (+SD) of wild type Arabidopsis thaliana seeds is shown. As a result of overlapping mass-transitions with isobaric phytosterols (Additional file 4: Table S2) the phytosterol species 18:2-sitosterol, 18:3-sitosterol and 18:2-stigmasterol lead to high false positive signals for 20:0/26:0, 20:1/26:0 and 20:0/26:1 wax ester species, respectively. [file 1746-4811-9-24-S3.pdf]

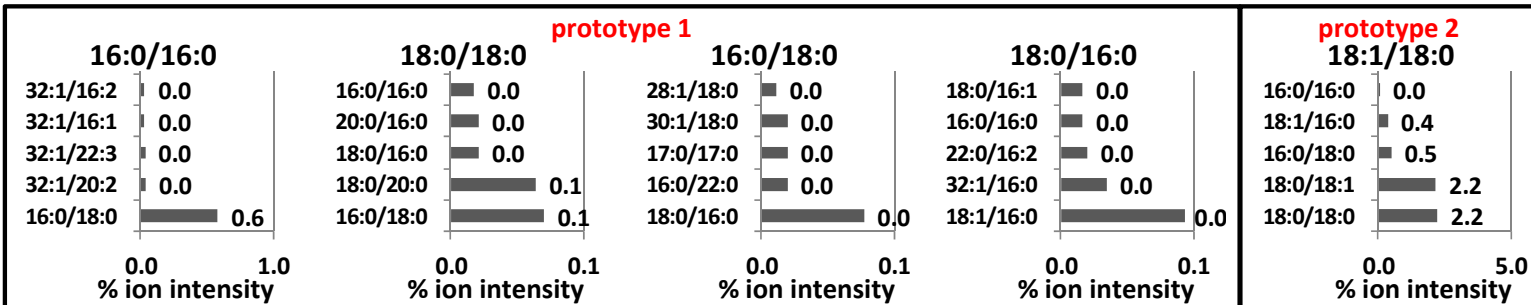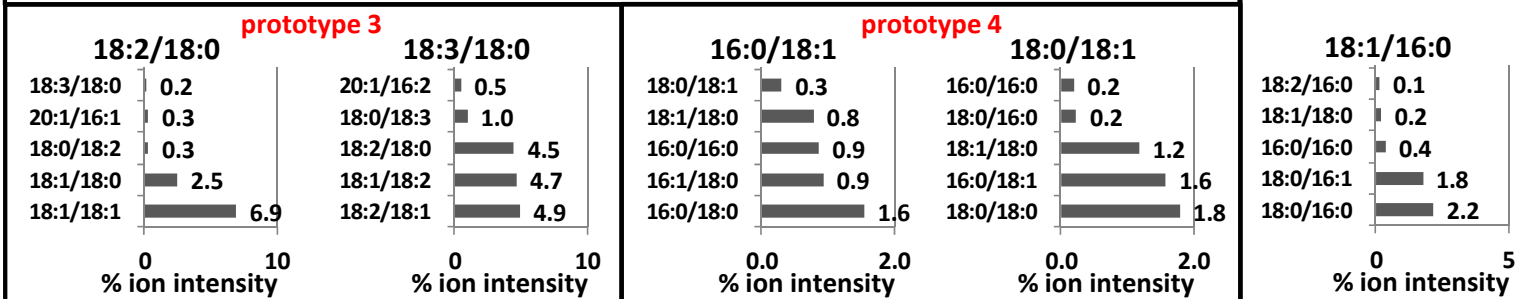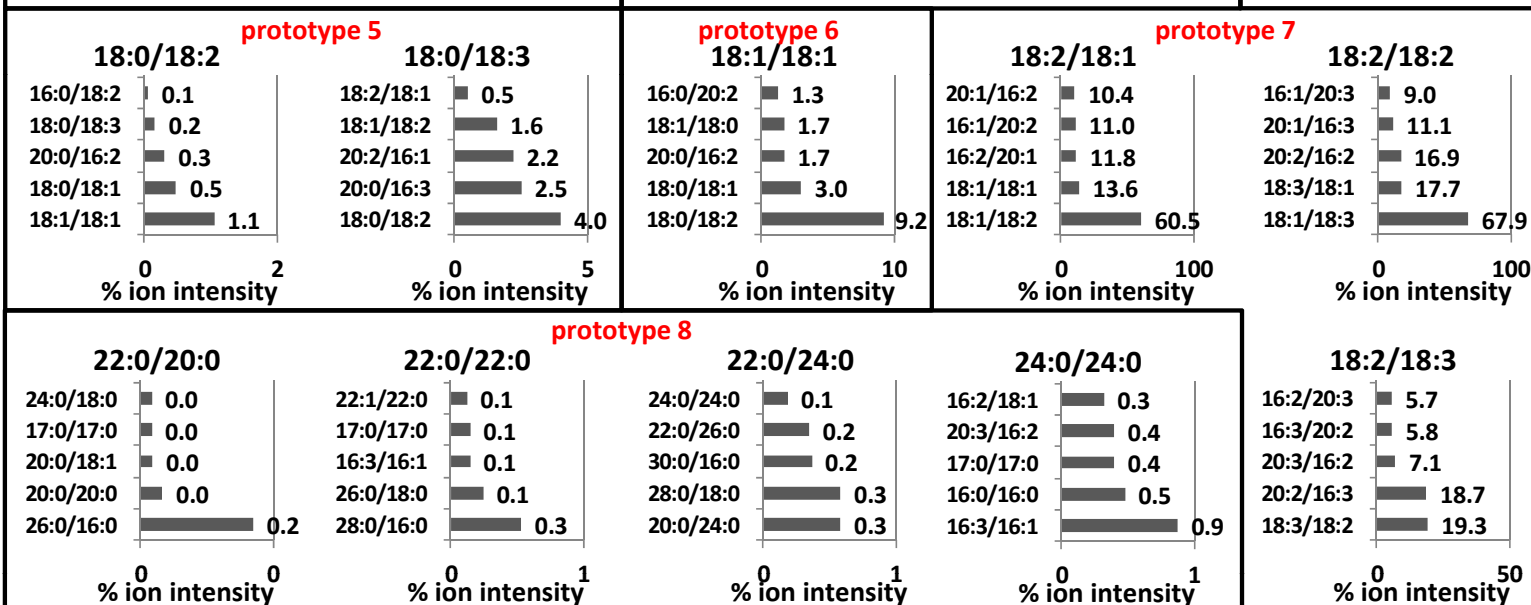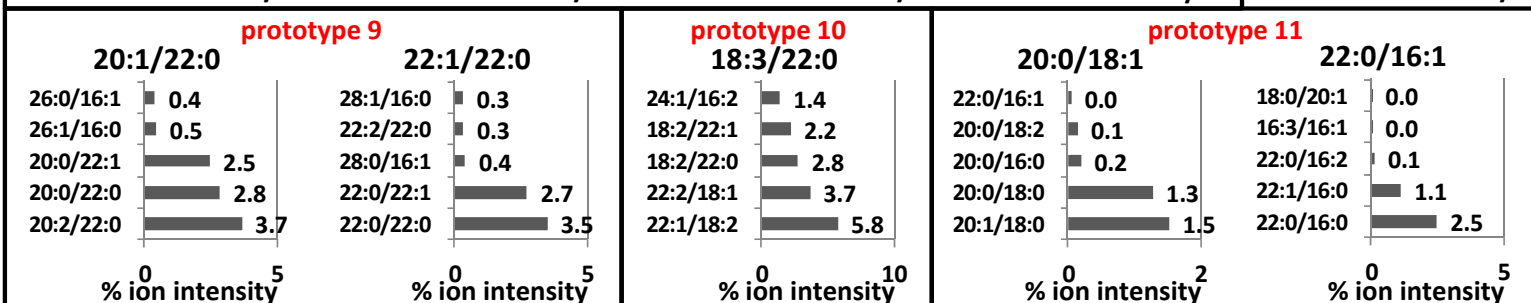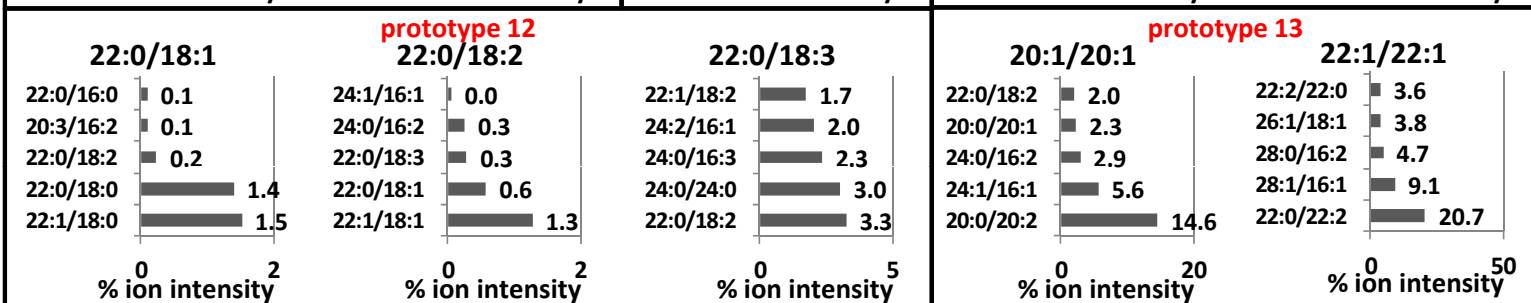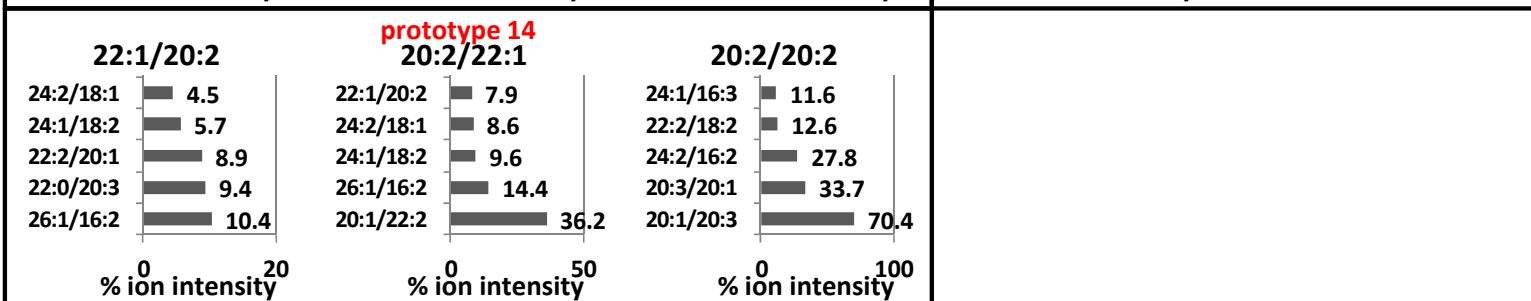

Supplement: Additional file 5: Figure S3 — MRM specificity for wax ester identification. The specificity of MRM transitions for wax ester detection was probed by analyzing single dilutions of 33 wax ester standards representing prototype groups 1–14. The five false positive signals of highest intensities (cps) from wax esters not present in the sample is expressed as % ion intensity of the positive signal from the analyzed wax ester species. False positive signals originate from detection of isobaric wax ester species and the C13 type II isotope effect. [file 1746-4811-9-24-S5.pdf]
