# Supplementary material for: Wax ester profiling of seed oil by nano-electrospray ionization tandem mass spectrometry
Source: Plant Methods. 2013 Jul 6;9:24. doi: 10.1186/1746-4811-9-24 (PMC3766222; doi:10.1186/1746-4811-9-24)
Supplement: Additional file 4: Table S2 — Wax ester molecular species that are isobaric with steryl esters. [file 1746-4811-9-24-S4.pdf]

MRM transition	Wax Ester	Steryl Ester
636.6/369.4	18:1/24:0	16:3-Cholesterol
638.6/369.4	18:0/24:0	16:2-Cholesterol
664.6/369.4	20:1/24:0	18:3-Cholesterol
664.7/395.2	18:0/26:1	16:2-Stigmasterol
664.7/397.2	18:1/26:0	16:3-Sitosterol
666.6/369.4	20:0/24:0	18:2-Cholesterol
666.7/397.2	18:0/26:0	16:2-Sitosterol
692.7/369.4	22:1/24:0	20:3-Cholesterol
692.7/395.2	20:0/26:1	18:2-Stigmasterol
692.7/397.2	20:1/26:0	18:3-Sitosterol
694.7/369.4	22:0/24:0	20:2-Cholesterol
694.7/397.2	20:0/26:0	18:2-Sitosterol
720.7/369.4	24:1/24:0	22:3-Cholesterol
720.7/395.2	22:0/26:1	20:2-Stigmasterol
720.7/397.2	22:1/26:0	20:3-Sitosterol
722.7/397.2	22:0/26:0	20:2-Sitosterol
722.8/369.4	24:0/24:0	22:2-Cholesterol
748.7/395.2	24:0/26:1	22:2-Stigmasterol
748.7/397.2	24:1/26:0	22:3-Sitosterol
750.7/397.2	24:0/26:0	22:2-Sitosterol
