# Supplementary material for: Wax ester profiling of seed oil by nano-electrospray ionization tandem mass spectrometry
Source: Plant Methods. 2013 Jul 6;9:24. doi: 10.1186/1746-4811-9-24 (PMC3766222; doi:10.1186/1746-4811-9-24)
Supplement: Additional file 6: Figure S4 — Determination of the method variation in % RSD from replicate extractions and replicate measurements. The % relative standard deviation (% RSD) from (B) five extraction replicates and (D) five replicate measurements of an individual transgenic line is shown. For the ten most abundant wax esters the mean (+SD) wax ester accumulation in mol% of total wax esters is depicted for (A) the replicate extractions and (C) the replicate measurements. [file 1746-4811-9-24-S6.pdf]

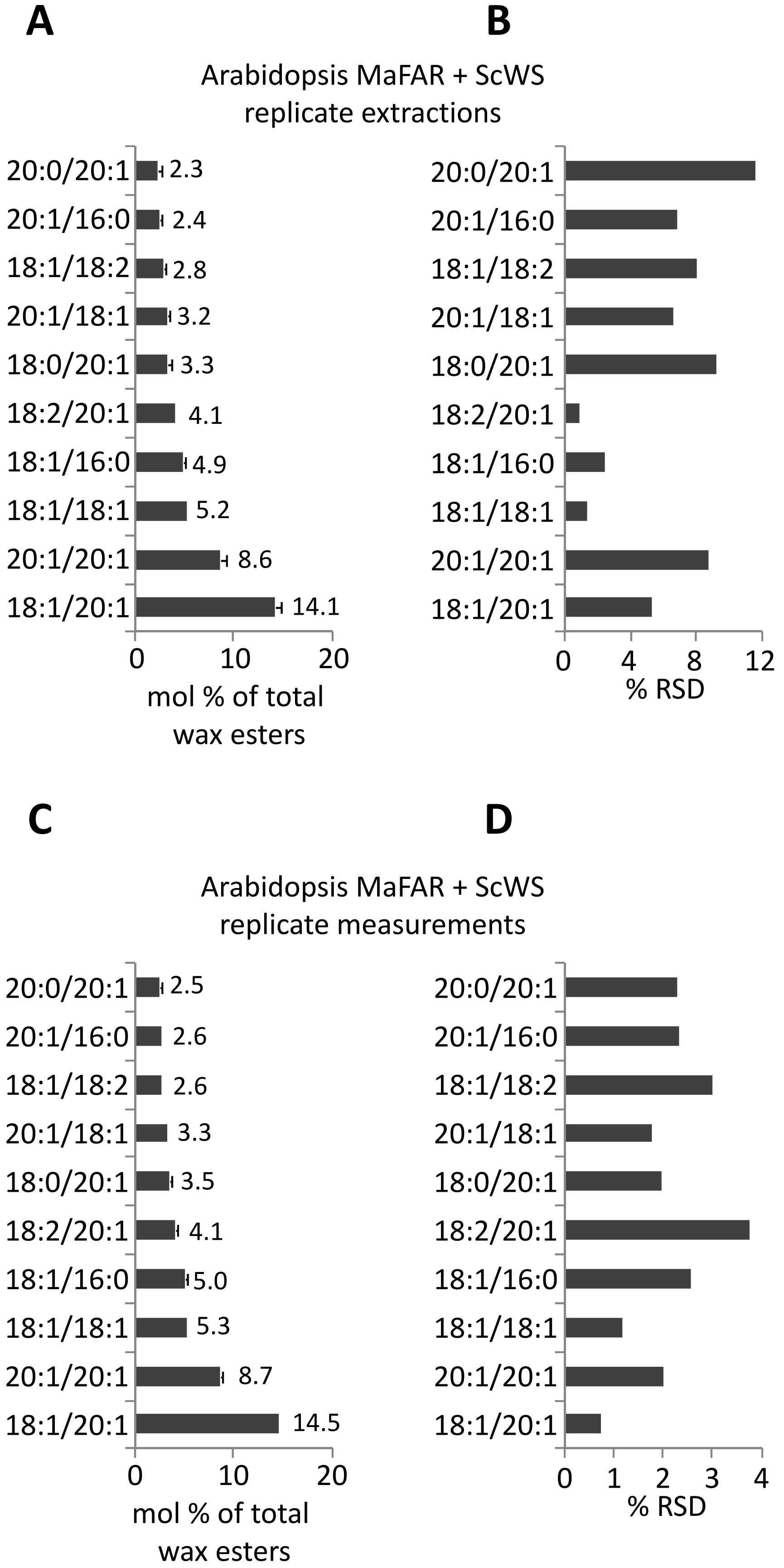
A
B
Arabidopsis MaFAR + ScWS
replicate extractions
20:0/20:1
20:1/16:0
18:1/18:2
20:1/18:1
18:0/20:1
18:2/20:1
18:1/16:0
18:1/18:1
20:1/20:1
18:1/20:1
2.3
2.4
2.8
3.2
3.3
4.1
4.9
5.2
8.6
14.1
0
10
20
mol % of total
wax esters
0
4
8
12
% RSD
C
D
Arabidopsis MaFAR + ScWS
replicate measurements
2.5
2.6
2.6
3.3
3.5
4.1
5.0
5.3
8.7
14.5
0
10
20
mol % of total
wax esters
0
1
2
3
4
% RSD
